# Risk factors for wheezing in infants born in Cuba

**DOI:** 10.1093/qjmed/hct143

**Published:** 2013-08-07

**Authors:** S. J. Venero-Fernández, R. Suárez-Medina, E. C. Mora-Faife, G. García-García, I. Valle-Infante, L. Gómez-Marrero, G. Abreu-Suárez, J. González-Valdez, D. Dania Fabró-Ortiz, H. Fundora-Hernández, A. Venn, J. Britton, A. W. Fogarty, THE HINASIC (Historia Natural de la Sibilancia en Cuba/National History of Wheezing in Cuba) Study Group

**Affiliations:** ^1^Instituto Nacional de Higiene, Epidemiología y Microbiología, Código Postal 10300, La Habana, Cuba, ^2^Hospital Universitario Pediátrico Docente Centro Habana, La Habana, Cuba, ^3^Hospital Pediátrico Docente “Juan Manuel Márquez”, La Habana, Cuba and ^4^Division of Epidemiology and Public Health, University of Nottingham, Clinical Sciences Building, City Hospital, Nottingham NG5 1PB, UK

## Abstract

**Background:** Cuba is a unique country, and despite limited economic development, has an excellent health system. However, the prevalence of asthma symptoms in children in Havana, Cuba, is unusually high.

**Aim:** As early life exposures are critical to the aetiology of asthma, we have studied environmental influences on the risk of wheezing in Cuban infants.

**Design:** Cross-sectional study.

**Methods:** A random sample of 2032 children aged 12–15 months living in Havana was selected for inclusion in the cohort. Data were collected using questionnaires administered by researchers.

**Results:** Of 2032 infants invited to participate, 1956 (96%) infants provided data. The prevalence of any wheeze was 45%, severe wheeze requiring use of emergency services was 30% and recurrent wheeze on three or more occasions was 20%. The largest adjusted risk factors for any wheeze were presence of eczema [odds ratio (OR) 2.09; 95% confidence interval (CI) 1.48–2.94], family history of asthma (OR 2.05; 95% CI 1.60–2.62), poor ventilation in the house (OR 1.99; 95% CI 1.48–2.67), attendance at nursery (OR 1.78; 95% CI 1.24–2.57), male sex (OR1.52; 95% CI 1.19–1.96) and the number of smokers in the house (*P* < 0.03 for trend), OR 1.64 (95% CI 1.17–2.31) for three or more smokers in the house compared to no smokers in the household.

**Conclusion:** We have identified several risk factors for any wheeze in young infants living in modern day Cuba. As the prevalence of smoking in the house is high (51%), intervention studies are required to determine effective strategies to improve infant health.

## Introduction

Asthma is a disease that often presents during the first decade of life, with an estimated global prevalence of wheezing in 6- to 7-year–old children of ∼12%.^1^ The prevalence of asthma and associated symptoms has increased substantially in the past five decades,^2^ often in parallel with economic development and urbanization.^3–5^ and increases in prevalence appear to be continuing in Africa, Latin America and parts of Asia^1^ where the rate of economic development is greatest. Therefore, environmental exposures are likely to be key components of the aetiology of asthma and epidemiological studies from differing societies and cultures have the potential to increase understanding of which environmental exposures consistently result in asthma globally, and which ones may be important within certain localized populations.

Cuba is a large island in the Caribbean with a unique cultural and historical experience, that has a system of universal health coverage,^6^ but in recent decades has experienced an economic blockade by the USA that has limited its economic development.^7^^,^^8^ There is concern that asthma prevalence in children may be particularly high in Cuba, with reported prevalences of wheezing in the past 12 months of 32% in boys aged 6–7 years living in Havana.^9^ As many of the risk factors for asthma are considered to occur in early life,^10^ a cohort of infants were recruited to attempt to identify environmental exposures that may be amenable to intervention in this population.

## Methods

### Study population

All children aged between 12 and 15 months who were living in Havana, Cuba between March 2010 and March 2011 and who attended one of the randomly selected 17 polyclinics, nested in four municipalities in Havana, Cuba, were eligible to be selected to participate in the study (Arroyo Naranjo, Cerro, Havana del Este, La Lisa). Individuals with a pre-existing diagnosis of neuropathy, myopathy, heart disease, genetic disease, such as cystic fibrosis, severe somatic malformations and those with limited life expectancy were excluded from the study. In total, 2195 infants were randomly selected to participate in the study proportionate numbers to each municipality population and after consent was obtained from the parents/guardians, the child was enrolled in the study. About 163 individuals were initially enrolled but subsequently found not to be eligible for inclusion in the study as they lived outside the polyclinic or municipality catchment area. The study protocol was approved by National Institute of Hygiene, Epidemiology and Microbiology, the local Havana Scientific Committee in Cuba and also by the University of Nottingham Medical School ethics committee in the UK.

### Data collection

The baseline data collection consisted of an interviewer-administered questionnaire that collated the responses from the parent/caretaker about prenatal and postnatal exposures of the child, their living environment and the medical history of the family. Specific questions focussed on paracetamol exposure and exposure to environmental tobacco smoke. Data on the height and weight at the time of the interview were also collected. The primary outcome was wheeze and used the Spanish translation of the ISAAC questionnaire ‘¿Ha tenido su bebé sibilancias o silbido, jipidos o ruidos en el pecho durante el primer año de vida?’ after piloting in the local community. Recurrent wheezing was characterized as wheeze on three or more occasions in the infant’s life, whereas the use of the emergency services to obtain treatment for wheezing was used to categorize severe wheeze. Where possible, a stool sample was also taken and analysed for parasite infection using direct method with eosin and Lugol solutions and concentration technique of Willis–Malloy using protocols from the Pedro Kouri Institute of Tropical Medicine, Havana.

## Data analysis

The data were entered into an electronic database, cleaned and checked for obvious errors or implausible values. All statistical analyses were carried out in Stata v12 (StataCorp, TX, USA) using the survey commands to allow for the clustered survey design. Univariate analyses were initially performed using logistic regression and crude odds ratios (ORs) and associated 95% confidence intervals (CIs) were computed for each exposure variable. Variables that were statistically significant in univariate analysis (*P* ≤ 0.05) were then entered into a mutually adjusted multivariable model and a step-wise modelling procedure followed to obtain a final model of only statistically significant (*P* ≤ 0.05) variables. Sex and number of smokers in the household were considered as *a priori* confounding factors. As many measures of smoke exposure, such as maternal, paternal and grandparental smoking were associated with increased wheeze in the univariate analysis; a composite measure of environmental tobacco exposure was computed as the total number of smokers in the household was included in all final models. No further *post hoc* modelling was performed on the dataset to maintain transparency and simplicity.

## Results

**Figure 1.**
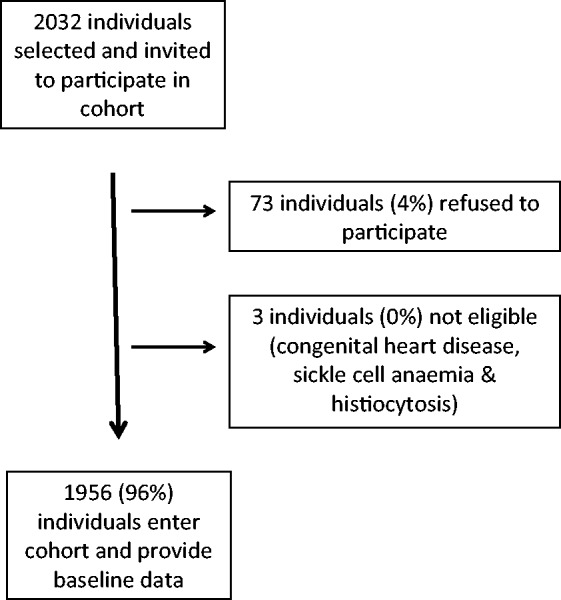
Flow diagram of participant recruitment into cohort.

The recruitment of individuals to the cohort is described in Figure 1. In summary, of 2032 individuals who were enrolled and eligible to enter the study, 1956 (96%) participated and provided the baseline data. The characteristics of this population are given in Table 1. Prevalence of any wheeze was 45%, severe wheeze requiring use of emergency services was 30% and recurrent wheezing on three or more occasions was 20%.

**Table 1:** Characteristics of study participants

Variable	Definition of category	Number (%) unless stated otherwise	Prevalence of any wheeze (%)	Univariate OR (95% CI) for any wheeze using survey commands
Mean age in months (SD)		13.1 (1.1)	n/a	1.09 (1.01–1.19) per month
Skin colour	White	916 (47)	378 (41)	1
	Mixed	798 (41)	384 (48)	1.32 (0.82–2.13)
	Black	242 (12)	110 (45)	1.19 (0.68–2.05)
Gender	Female	939 (48)	370 (39)	1
	Male	1017 (52)	502 (49)	1.50 (1.12–2.01)
Municipality	Habana del Este	642 (33)	224 (35)	1
	Cerro	374 (19)	162 (43)	1.43 (1.14–1.79)
	La Lisa	282 (14)	149 (53)	2.09 (1.65–2.66)
	Arroyo Naranjo	658 (34)	337 (51)	1.96 (1.54–2.50)
Highest educational status of mother	Primary	17 (1)	9 (53)	1.42 (0.76–2.65)
	Secondary	431 (22)	206 (48)	1.16 (0.84–1.59)
	Pre-university	1157 (59)	511 (44)	1
	University	351 (18)	146 (42)	0.90 (0.62–1.30)
Mother with paid work	No	780 (40)	370 (47)	1
	Yes	1176 (60)	502 (43)	0.83 (0.74–0.92)
Household income (pesos)	>3000	35 (2)	14 (40)	0.91 (0.37–2.24)
	2000–3000	48 (2)	25 (52)	1.48 (0.31–7.01)
	1001–1999	207 (11)	97 (47)	1.20 (0.81–1.80)
	500–1000	955 (49)	404 (42)	1
	<500	711 (36)	332 (47)	1.19 (0.83-1.72)
Any siblings	No	818 (42)	342 (42)	1
	Yes	1138 (58)	530 (47)	1.21 (0.89–1.65)
Any older siblings	No	850 (43)	356 (42)	1
	Yes	1106 (57)	516 (47)	1.21 (0.95–1.54)
Number of older siblings	0	850 (43)	356 (42)	1
	1	661 (34)	315 (48)	1.26 (0.88–1.82)
	2	289 (15)	129 (45)	1.12 (0.92–1.35)
	3	104 (5)	44 (42)	1.02 (0.58–1.78)
	4+	52 (3)	28 (54)	1.62 (0.72–3.66)
Mean age of mother at birth in years (SD)		26.7 (6.2)	n/a	0.98 (0.95–1.01)
		*N* = 1955		per year
Any wheeze in lifetime?	Yes	872 (45)	–	–
	No	1084 (55)		
Severe wheeze requiring emergency services in lifetime?	Yes	590 (30)	–	–
	No	1366 (70)		
Recurrent wheeze (three or more episodes)	Yes	386 (20)	–	–
	No	1570 (80)		

After mutual adjustment for confounding, the independent risk factors for any wheeze in the first year of life are presented in Table 2. The largest risk factors for any wheeze were self-reported eczema (OR 2.09; 95% CI 1.48–2.94), a positive family history of asthma (OR 2.05; 95% CI 1.60–2.62), poor ventilation (OR 1.99; 95% CI 1.48–2.67), nursery attendance (OR 1.78; 95% CI 1.24–2.57) and male sex (OR 1.52; 95% CI 1.19–1.96) and the number of smokers in the house (*P* < 0.03 for trend), OR 1.64 (95% CI 1.17–2.31) for three or more smokers in the house compared to no smokers in the household. The Habana del Este municipality has a consistently lower risk of any wheeze, recurrent wheezing and severe wheeze than the other municipalities which was not explained by any of the variables adjusted for in the analysis. There was no association between paracetamol administration to the infant in the first year of life (76% prevalence) and risk of wheezing (OR 1.20; 95% CI 0.83–1.74 in the univariate analysis).

**Table 2:** Multivariate analysis of exposures and risk of any wheeze

Variable	Definition of category	Number	Adjusted OR (95% CI) for any wheeze
Municipality (%)	Habana del Este	642 (33)	1
	Cerro	374 (19)	1.73 (1.31–2.29)
	La Lisa	282 (14)	2.11 (1.64–2.72)
	Arroyo Naranjo	658 (34)	2.02 (1.56–2.63)
Self-reported eczema	No	1284 (66)	1
	Yes	672 (34)	2.09 (1.48–2.94)
Family history of asthma (%)	No	917 (47)	1
	Yes	1039 (53)	2.05 (1.60–2.62)
Ventilation of house (%)	Good	1535 (78)	1
	Regular	307 (16)	1.02 (0.56–1.86)
	Poor	114 (6)	1.99 (1.48–2.67)
Child attended daycare/nursery (%)	No	1685 (86)	1
	Yes	271 (14)	1.78 (1.24–2.57)
Number of smokers in house (%)	0	952 (49)	1
	1	494 (25)	1.11 (0.88–1.39)
	2	340 (17)	1.17 (0.62–2.18)
	≥3	170 (9)	1.64 (1.17–2.31)
			*P*_trend_ = 0.03
Sex (%)	Female	939 (48)	1
	Male	1017 (52)	1.52 (1.19–1.96)
Insect sting allergy (%)	No	933 (48)	1
	Yes	1022 (52)	1.45 (1.07–1.97)
Infant’s room walls painted after birth	No	1713 (88)	1
	Yes	243 (12)	1.40 (1.04–1.89)
Any siblings (%)	No	1138 (58)	1
	Yes	818 (42)	1.17 (1.02–1.33)
Mean age in months (range)		13.1 (12–15)	1.13 (1.01–1.28) per month
Infant’s mean height at birth per centimetre (SD)	50.28 (2.38)	10.52 (1.56)	0.95 (0.92–0.98)
Mother with paid work (%)	No	780 (40)	1
	Yes	1176 (60)	0.86 (0.75–0.99)

1950 individuals provided complete data for analysis

## Discussion

This is the first population-based study to investigate exposures that are associated with increased risk of wheeze in Havana, Cuba, a relatively unique environment that is considered to possibly have a high prevalence of wheezing in children. Our initial cross-sectional analysis of the baseline data at the age of 12–15 months has identified a number of risk factors for any wheeze in these individuals. The exposures that are associated with an increased risk of any wheeze include family history of asthma, self-reported eczema, poor ventilation, male gender, smaller height at birth, the presence of other siblings, attendance at daycare facilities, living with smokers in the household and living in either Cerro, Arroyo Naranjo or La Lisa municipalities of Havana City.

The strengths of these dataset include the recruitment of four areas in Havana that are considered to be representative of the population of Havana, and the high response rate of 96% of those who were eligible and invited to participate, giving confidence that the results are unlikely to be susceptible to response bias. The data were collected by staff at the local participating polyclinics who ensured that all questions were answered and hence there is little missing data. The questions on exposures that were considered to be potentially important with regard to the aetiology of asthma in this population were devised by Cuban epidemiologists familiar with the environment, and we were thus able to test hypotheses that had been formulated locally. As emergency medical treatment is easily accessible in Havana and mothers are encouraged to have a low threshold for accessing this care, the latter category of severe wheeze may not be generalizable to other populations.

As we were trying to identify important risk factors for wheezing in a relatively unstudied population, we necessarily have tested many exposures leading to the risk that some of our apparently statistically significant associations are artefacts of multiple hypothesis testing. The cross-sectional nature of the study will not permit causality to be identified, simply associations and in particular, is unable to exclude the possibility of reverse causality or residual confounding by unmeasured exposures. As the phenotype of asthma takes a while to develop, it is important to clarify that this is a study of risk factors for wheezing in young children, rather than simply asthma. This is particularly important in this population of infants aged 12–15 months, as much wheeze is likely to be viral in nature,^11^ which would explain the increased risk of wheeze in those who are attending daycare facilities or have other siblings—these individuals being more likely to encounter other children and hence acquire viral infections which manifest themselves as wheeze and other respiratory symptoms. Another limitation is the inability to collect data on all potential factors that may modify risk of wheezing and associated allergic disease in young children, such as faecal microbiota^12^ and allergen exposure,^13^ which were beyond the scope of the resources available to us. We were, however, able to collect data on faecal parasite infection in a subgroup of 712 individuals, but this was very low in prevalence (4%) and hence not surprisingly, was not an important determinant of wheezing in our population in this age group. Finally, in a survey such as this, differential measurement error may result in significant associations for exposures with little measurement error such as age, compared to those with more measurement error, e.g. presence of air pollution near the house^14^ which consequently have a higher variance.

Although we were not surprised to identify that consistent with the literature, family history of asthma,^15^ male sex,^16^ concurrent eczema and environmental tobacco smoke^17^ were risk factors for any wheeze in young children, our data did generate some new observations that warrant critical consideration. The persistent observation of insect skin allergy being associated with increased risk of any wheeze, suggests a potential cause of cutaneous sensitization associated with respiratory symptoms in young children that is worthy of future study. One limitation of our data is that we are not aware which insects in particular may drive this association and this will need further consideration in future studies as this may provide a reversible exposure of wheeze and asthma if a causal association was demonstrated.

It is unclear why living in Habana del Este should be associated with a substantially lower risk of wheezing than other areas of Havana. Habana del Este is situated in a coastal location with relatively less heavy industry and a lower density of motor traffic than the other municipalities that contributed to the study population, suggesting that urbanization and pollution may contribute to the geographical differences in risk of wheezing. Future studies of the risk of wheezing in Havana would benefit from objective measures of air pollution to test the hypothesis that this may contribute to the disease burden of asthma in this setting.

In conclusion, we report data from a cohort of infants aged 12–15 months living in Havana which has a high frequency of any wheeze, with 45% having any wheeze reported since birth. The location of residence is an important risk factor for wheezing, as are others such as exposure to environmental tobacco smoke, the presence of poor ventilation in the house, the presence of siblings and smaller mean height at birth. Interventions to reduce environmental tobacco exposure in this population are required.
